# An energy and information analysis method of logic gates based on stochastic thermodynamics

**DOI:** 10.1093/pnasnexus/pgae365

**Published:** 2024-08-26

**Authors:** Xiaohu Ge, Muyao Ruan, Xiaoxuan Peng, Yong Xiao, Yang Yang

**Affiliations:** School of Electronic Information and Communications, Huazhong University of Science and Technology, Wuhan 430074, Hubei, China; International Joint Research Center of Green Communications and Networking, Huazhong University of Science and Technology, Wuhan 430074, Hubei, China; School of Electronic Information and Communications, Huazhong University of Science and Technology, Wuhan 430074, Hubei, China; International Joint Research Center of Green Communications and Networking, Huazhong University of Science and Technology, Wuhan 430074, Hubei, China; School of Electronic Information and Communications, Huazhong University of Science and Technology, Wuhan 430074, Hubei, China; International Joint Research Center of Green Communications and Networking, Huazhong University of Science and Technology, Wuhan 430074, Hubei, China; School of Electronic Information and Communications, Huazhong University of Science and Technology, Wuhan 430074, Hubei, China; 5G Verticals Innovation Laboratory, Huazhong University of Science and Technology, Wuhan 430074, Hubei, China; IoT Thrust, Information Hub, Hong Kong University of Science and Technology (Guangzhou), Guangzhou 511458, Guangdong, China

**Keywords:** information thermodynamics, information energy ratio, nonequilibrium information processing, digital circuit

## Abstract

To reduce the energy consumption of logic gates in digital circuits, the size of transistors approaches the mesoscopic scale, e.g. sub-7 nanometers. However, existing energy consumption analysis methods exhibit various deviation for logic gates when the nonequilibrium information processing of mesoscopic scale transistors with ultra-low voltages is analyzed. Based on the stochastic thermodynamics theory, an information energy ratio method is proposed for the energy consumption estimation of XOR gates composed of mesoscopic scale transistors. The proposed method provides a new insight to quantify the transformation between the information capacity and energy consumption for XOR gates and extending to other logic gates. Utilizing the proposed analysis method, the supply voltage of the parity check circuit can be optimized by numerical simulations without expensive and complex practical measurements. The information energy ratio is the first analytical method to quantify the energy and information transformation of logic gates at the mesoscopic scale.

**Significance Statement**

This research is crucial for further understanding factors that affect energy consumption in digital logic circuits by addressing challenges in mesoscopic-scale transistors. The authors propose a groundbreaking method based on stochastic thermodynamics to determine the ratio between information capacity and energy consumption. This provides a unique perspective on the interplay between the information capacity and energy consumption for logic gates. The proposed method facilitates the optimization of supply voltage through numerical simulations, reducing the need for expensive practical measurements. This pioneering analytical tool represents a significant leap in quantifying energy and information capacity transformation within logic gates.

With the fast-growing deployment of the 5th-generation (5G) mobile communication, artificial intelligence (AI) and big data systems, the massive data need to be processed by digital signal processing circuits. The energy consumption of digital signal processing circuits is increased quickly in 5G mobile communication, AI, and big data systems (1, 2). Digital signal processing circuits are composed of three types of basic logic gates, i.e. AND, NOT, and XOR gates, all of which are made up of transistors (3). Thanks to the recent advancement of circuit technologies, the size of transistors is coming into the mesoscopic scale, e.g. sub-7 nanometers (nm), and continuing increases have been provided in computing capability (4). However, when the sub-7 nm transistor technology is adopted, digital logic circuits are inevitably becoming more and more susceptible to the thermal noise due to the aggressive voltage and gate length scaling (5), especially at the mesoscopic scale. Traditional energy consumption analytical methods of digital logic circuits take into account the thermal noise from phenomenological approaches (6). Hence, traditional energy consumption analytical methods are difficult to reveal the energy and information transformation mechanism of digital circuits due to the thermal fluctuations of nonequilibrium information processing at the mesoscopic scale (7). To overcome the faultiness of traditional energy consumption analytical methods, the stochastic thermodynamics is introduced to analyze the nonequilibrium information processing of transistors at the mesoscopic scale (8). Recently, the information thermodynamic theory-based energy consumption models were explored for logic gates (9). However, it is a great challenge to design an analysis method to clarify the transformation ratio between the information and energy with a specific process at logic gates, which can be used to optimize the energy consumption of logic gates, such as XOR operations at XOR gates adopted the complementary metal-oxide-semiconductor (CMOS) technology. Considering the complexity of nonequilibrium information processing process of transistors at the mesoscopic scale, it is a great challenge to propose an analytical method of information and energy transformation for logic gates and digital circuits.

To describe the nonequilibrium information process in digital circuits, the information thermodynamic theory has been utilized to explore the thermal, work, and entropy of digital signal processing (7, 10–13). Freitas et al. (7) established a stochastic thermodynamic model to investigate the irreversible entropy production of nonlinear electronic circuits subject to thermal noise. The mismatch entropy production was proposed to differentiate the entropy of the actual input distribution and the entropy of the optimal input distribution in a thermodynamic system (10). Recently, the energy dissipation of digital circuits has been investigated by the mismatch entropy production and Landauer’s principle (11). In particular, Koski et al. (12) characterized the thermodynamic entropy production model of single-electron transistors and derived a generalized fluctuation theorem. Wimsatt et al. (13) analyzed the physical factors that influence the energy consumption of computation systems, including the computing rate, computing error rate, storage stability, circuit modularity, etc. Sadasivan Shankar et al. (14) evaluated the efficiency of an ideal computing architecture using thermodynamics and quantum mechanics principles, which is applicable to both large computing systems and single switches.

Most existing studies on digital circuits focus on relatively simple scenarios extended from Landauer’s principle. The interaction between information capacity and energy consumption of logic gates is one of the most basic studies for the design of digital logic circuits. However, the study investigating the interaction between information capacity and energy consumption in logic gates, particularly considering the influence of thermal noise at the mesoscopic scale, is rare in the open literature. Motivated by the above gaps, in this article, we first propose an analysis method to quantify the transformation ratio between the information capacity and energy consumption for logic gates at the mesoscopic scale. The proposed analysis method can be extended to other logic gates and circuits by changing the combination of transistors and adjusting the linear relationship between energy levels and voltages. The key contributions of this article are briefly summarized as follows:

1. Based on the stochastic thermodynamics and information theory, an analysis method, i.e. the information energy ratio, is first modeled to reveal the interplay between the information capacity and energy consumption of XOR gates composed of mesoscopic scale transistors. Moreover, the proposed analysis method can be extend to other logic gates by changing the combination of transistors and adjusting the linear relationship between energy levels and voltages. The proposed analysis method provides a new insight to quantify the transformation ratio between the information capacity and energy consumption for logic gates. Furthermore, an upper bound of the information energy ratio of XOR gates is derived.
2. Based on the proposed analysis method, the information energy ratio is used to analyze the transformation ratio between the information capacity and energy consumption for the parity check circuit.
3. Utilizing the proposed analysis method to simulate the information energy ratio of the parity check circuit adopting the 7 nm semiconductor process supply voltage, simulation results show that the information energy ratio of the parity check circuit is improved by 266% when the supply voltage is set to a chosen value. These results indicate that the proposed analysis method can be used to optimize the energy consumption of digital signal processing circuits by numerical simulations without expensive and complex practical measurements.

## Energy consumption of XOR gates

**Fig. 1.**
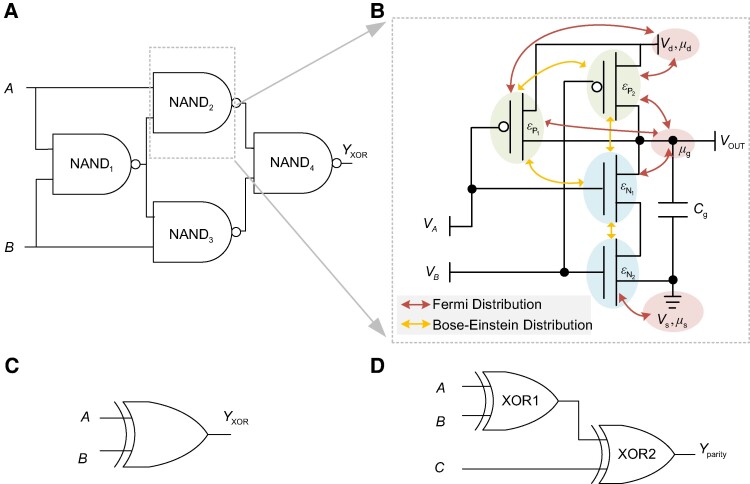
Models of XOR gates and the parity check circuit. a) Circuit diagram of XOR gates. The two inputs of the XOR gate are labeled as *A* and *B*, and the output is $Y_{\mathrm{XOR}}$. b) Kinetic diagram of the gate $\mathrm{NAN}D_{r}$, where r∈{1,2,3,4}. Each NAND gate is composed of P-type transistors and N-type transistors. Transistors and electrodes are expressed as different energy levels $\varepsilon_{\;j}$ and chemical potentials $\mu_{i}$, respectively, with $j\in \{P_{1},P_{2},N_{1},N_{2}\}$, i∈{d,s,g}. $V_{A}$ and $V_{B}$ are the voltages of double-inputs of the NAND gate. The output voltage of $\mathrm{NAN}D_{r}$ is denoted as $V_{\mathrm{out}}$ and the load capacitance is $C_{g}$. The supply voltage is $V_{d}$, and the voltage to ground is $V_{s}$. c) Schematic diagram of XOR gates. d) The parity check circuit can be composed of two XOR gates shown as XOR1 and XOR2.

In this article, an XOR gate is composed of four single-electron NAND gates, as shown in Fig. 1a. The dynamic electron transfer process of each NAND gate is illustrated in Fig. 1b. Fig. 1c shows the schematic diagram of XOR gates. XOR gates can be used to compose a variety of commonly used circuits, such as the parity check circuit, which is shown in Fig. 1d. As shown in Fig. 1d, the parity check circuit is composed of two XOR gates connected in series. Three inputs of the parity check circuit are represented as *A*, *B*, and *C* and the output is represented as $Y_{\mathrm{parity}}$, A,B,C∈{0,1}, $Y_{\mathrm{parity}}\in Y_{\mathrm{XOR}}$, which is the all output space of XOR gates. In this way, the physical operation process of XOR gates can be abstracted as the electron transfer process. The derivation of the energy consumption of NAND gates is given in Supplementary Material, Appendix S1. The energy consumption of XOR gates is composed of the energy consumption of four NAND gates, which is expressed as


(1) $$W_{\mathrm{XOR}}(\tau )=\sum_{r=1}^{4}W_{\mathrm{NAND}}^{r}(\tau ),$$

where $W_{\mathrm{NAND}}^{r}(\tau )$ is the energy consumption of ${\text{NAND}}_{r}$ when the propagation delay is *τ*, r∈{1,2,3,4}. The propagation delay is taken as the moment when the difference between the output voltage and the expected voltage exceeds a certain threshold.

**Fig. 2.**
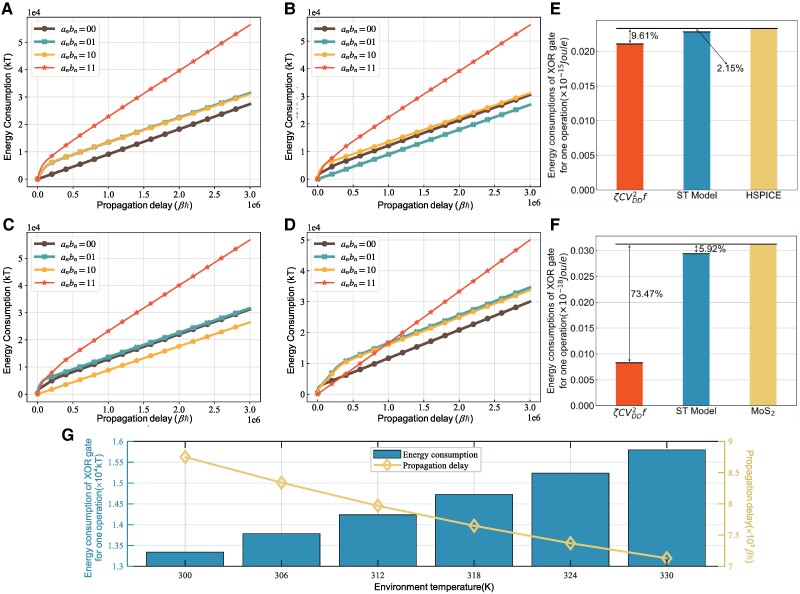
Energy consumption of XOR gates. a) The energy consumption curve of XOR gates when the previous input symbol is $a_{n-1}b_{n-1}=00$. b) The energy consumption curve when the previous input symbol is $a_{n-1}b_{n-1}=01$. c) The energy consumption curve when the previous input symbol is $a_{n-1}b_{n-1}=10$. d) The energy consumption curve when the previous input symbol is $a_{n-1}b_{n-1}=11$. e) Energy consumption of XOR gates for one operation with respect to $\zeta CV_{\mathrm{DD}}^{2}f$, the ST method and the simulation results based on HSPICE. f) Energy consumption of XOR gates for one operation with respect to $\zeta CV_{\mathrm{DD}}^{2}f$, the ST method and the 0.34 nm graphene side-wall edge gated MoS2 transistor. g) The energy consumption and propagation delay of XOR gates for one operation with different environment temperatures.

Assuming that input sequences are governed by the uniform distribution, Fig. 2a–d shows the energy consumption of XOR gate as a function of time (unit is βℏ, β=1/kT, where *k* is the Boltzmann coefficient, *T* is the temperature, ℏ is the reduced Planck constant. *kT* is the unit energy, $kT\approx 4.14\times 10^{-21}J$ when T=300K). when the time step is n−1, the input symbols $a_{n-1}b_{n-1}$ are configured as {00,01,10,11}, respectively. $a_{n}b_{n}$ are the input symbols at the time step *n*. In Fig. 2a, the curves when the input symbols at the time step *n* are 01 and 10 show a similar increasing trend, the curve corresponding to $a_{n}b_{n}=11$ shows the largest energy consumption, and the curve corresponding to $a_{n}b_{n}=00$ exhibits the lowest energy consumption. In Fig. 2b, the curves corresponding to $a_{n}b_{n}\in \{01,10\}$ show a similar increasing trend, the curve corresponding to $a_{n}b_{n}=00$ shows the largest energy consumption, and the curve corresponding to $a_{n}b_{n}=01$ exhibits the lowest energy consumption. In Fig. 2c, the curves corresponding to $a_{n}b_{n}\in \{00,01\}$ show a similar increasing trend, the curve corresponding to $a_{n}b_{n}=00$ shows the largest energy consumption, and the curve corresponding to $a_{n}b_{n}=10$ exhibits the lowest energy consumption. In Fig. 2d, the curves when the input symbols at the time step *n* are 01 and 10 show a similar increasing trend, the curve corresponding to $a_{n}b_{n}=11$ shows the largest energy consumption when the propagation delay is larger than 1.0 βℏ, and the curve corresponding to $a_{n}b_{n}=00$ exhibits the lowest energy consumption.

Considering that different previous inputs result in different voltages of capacitors in XOR gates, the energy consumption of XOR gates depends not only on the present input but also on the previous input. A string of double-inputs sequence streams with the length *M* is represented as $X_{\mathrm{in}}=\{a_{1}b_{1},a_{2}b_{2},\ldots$$a_{n-1}b_{n-1},a_{n}b_{n},\ldots ,a_{M}b_{M}\}$, 1≤n≤M, which are located at two inputs of XOR gates described as *A* and *B*, respectively. Without loss of generality, the set space of $a_{n}b_{n}$ is denoted as $a_{n}b_{n}\in \{00,01,10,11\}$. When four states of input at the previous time step are configured as {00,01,10,11}, the energy consumption of XOR gates can be calculated as follows


(2) $$E_{\mathrm{XOR}}^{\mathrm{diss}}=\left(\begin{array}{cccc} E_{00\to 00} & E_{00\to 01} & E_{00\to 10} & E_{00\to 11}\\ E_{01\to 00} & E_{01\to 01} & E_{10\to 10} & E_{10\to 11}\\ E_{10\to 00} & E_{10\to 01} & E_{10\to 10} & E_{10\to 11}\\ E_{11\to 00} & E_{11\to 01} & E_{11\to 10} & E_{11\to 11} \end{array}\right),$$

where $E_{a_{n-1}b_{n-1}\to a_{n}b_{n}}$ is the consumed energy when the previous input symbol is $a_{n-1}b_{n-1}$ at the time step n−1 and the current input symbol is $a_{n}b_{n}$ at the time step *n*. The input state transition matrix is


(3) $$P_{\mathrm{XOR}}^{\mathrm{trans}}=\left(\begin{array}{cccc} {\;p}_{00\to 00} & {\;p}_{00\to 01} & {\;p}_{00\to 10} & {\;p}_{00\to 11}\\ {\;p}_{01\to 00} & {\;p}_{01\to 01} & {\;p}_{01\to 10} & {\;p}_{01\to 11}\\ {\;p}_{10\to 00} & {\;p}_{10\to 01} & {\;p}_{10\to 10} & {\;p}_{10\to 11}\\ {\;p}_{11\to 00} & {\;p}_{11\to 01} & {\;p}_{11\to 10} & {\;p}_{11\to 11} \end{array}\right),$$

where $P_{a_{n-1}b_{n-1}\to a_{n}b_{n}}$ is the transition probability when the previous input symbol is $a_{n-1}b_{n-1}$ at the time step n−1 and the current input symbol is $a_{n}b_{n}$ at the time step *n*. Based on Eqs. 2 and 3, the average energy consumption of one operation at the XOR gate is given by


(4) $$W_{\mathrm{XOR}}=\sum_{a_{n-1}b_{n-1}}\sum_{a_{n}b_{n}}{\left(E_{\mathrm{XOR}}^{\mathrm{diss}}\cdot P_{\mathrm{XOR}}^{\mathrm{trans}}\right)}_{a_{n-1}b_{n-1},a_{n}b_{n}}.$$

The traditional energy consumption analysis method of XOR gates due to switching activities is expressed as


(5) $$P_{\mathrm{sw}}=\zeta CV_{\mathrm{DD}}^{2}f,$$

where *ζ* is the switching coefficient, *C* is the load capacitance, $V_{\mathrm{DD}}$ is the supply voltage, and *f* is the operating frequency (15, 16). When the technology of transistors is adopted to be 7 nm, the physical gate length is 54 nm. The equivalent load capacitance per unit gate length is $C=3\times 10^{-18}$ farad/nm (17), thus $C_{g}=1.62\times 10^{-16}$ farad. The rest of simulation parameters are configured as ζ=0.2 and $V_{d}=0.4$ Volt, $V_{T}=kT/q\approx 0.026$ Volt is the thermal noise voltage, i.e. the unit voltage of the supply voltage. *q* is the charge of the electron. Fig. 2e shows the energy consumption of XOR gates for one operation with respect to the traditional method, the ST method, and the hierarchical simulation program for integrated circuits emphasis (HSPICE) method (18). As shown in Fig. 2e, the deviation among three methods is within 10% at 7 nm CMOS technology. When the result of HSPICE method is configured as the benchmark, the deviation of the ST method is less than the deviation of the traditional method in the energy consumption estimation of XOR gates. When the gate size of transistors is 0.34 nm and the other parameters are configured as $V_{d}=5V_{T}$, $C_{g}=1.02\times 10^{-18}$ farad, and ζ=0.2, Fig. 2f shows the energy consumption of XOR gates for one operation with respect to the traditional method, the ST method, and the experimental data from the graphene side-wall edge gated MoS2 transistor (19), where the XOR gate based on the graphene side-wall edge gated MoS2 transistor consists of 16 transistors. Simulation results in Fig. 2f show that the deviation between the traditional method and the graphene side-wall edge gated MoS2 transistor is 73.47%. Therefore, the traditional method cannot used for analyzing the energy consumption of XOR gates at the sub-7 nm scale. Simulation results in Fig. 2f validate that the deviation between the ST method and the graphene side-wall edge gated MoS2 transistor is 5.92%. Hence, the ST method can still be used for analyzing the energy consumption of XOR gates at the sub-7 nm scale.

Moreover, the energy consumption and propagation delay of XOR gates for one operation with different environment temperatures are illustrated in Fig. 2g. When the environment temperature is increased, Fig. 2g shows that the energy consumption is increased and the propagation delay is decreased. The electron transfer rate of the XOR gate increases with the increase of environment temperature. As a consequence, the increased electron transfer rate causes an increase in the drain current of the XOR gate. In the end, the energy consumption of XOR gates is increased. Moreover, the time reaching the steady state is reduced in the electron transfer process of XOR gates when the electron transfer rate is increased. Therefore, the propagation delay of XOR gates is decreased in Fig. 2g.

## Information capacity of XOR gates

**Fig. 3.**
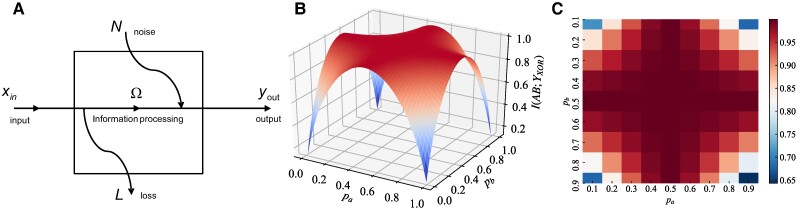
Schematic diagram of the information processing process and the mutual information of XOR gate with respect to the input distribution. a) Schematic diagram of the information processing process. b) The three-dimensional perspective. c) the heatmap projection on the ${\;p}_{a}$–${\;p}_{b}$ plane.

Figure 3a illustrates the information processing process, wherein the information processing module can be an algorithm, an integrated circuit, or a simple logic gate. In Fig. 3a, the input symbol is $x_{\mathrm{in}}$ which belongs to $X=\{x_{1},x_{2},\ldots x_{\mathrm{in}},\ldots ,x_{K}\}$ and the output symbol is $y_{\mathrm{out}}$ which belongs to $Y=\{y_{1},y_{2},\ldots y_{\mathrm{out}},\ldots ,y_{K}\}$.

When the information processing module is replaced by a XOR gate in Fig. 1c, the input symbols of information processing process are replaced by the inputs of XOR gate, i.e. *A* and *B* and the output symbol of information processing process are replaced by the output of XOR gate, i.e.$Y_{\mathrm{XOR}}$. Based on the threshold voltage judgment criterion, the input logic states and the output logic states are mapped as


(6a) $$\begin{array}{rl} A & =\left\{ \begin{array}{cc} 0 & V_{\mathrm{in}}=0\\ 1 & V_{\mathrm{in}}=V_{d} \end{array}\right., \end{array}$$

(6b) $$\begin{array}{rl} B & =\left\{ \begin{array}{cc} 0 & V_{\mathrm{in}}=0\\ 1 & V_{\mathrm{in}}=V_{d} \end{array}\right., \end{array}$$

(6c) $$\begin{array}{rl} Y_{\mathrm{XOR}} & =\left\{ \begin{array}{cc} 0 & V_{\mathrm{out}}<\left(1-\alpha \right)V_{d}\\ 1 & V_{\mathrm{out}}\geq \alpha V_{d}\\ \emptyset & \mathrm{otherwise} \end{array}\right., \end{array}$$

where $V_{\mathrm{in}}$ is the voltage of input and $V_{\mathrm{out}}$ is the voltage of output, *α* is the threshold factor. When the output voltage is higher than or equal to $\alpha V_{d}$, the output symbol is marked as the logic 1. When the output voltage is lower than $(1-\alpha )V_{d}$, the output symbol is marked as the logic 0. Otherwise, the output symbol is marked as ∅. The derivation of the mutual information of XOR gates is given in Supplementary Material, Appendix S2.

Figure 3b and c simulates the mutual information of XOR gate with respect to the input distribution. Fig. 3b presents the mutual information of XOR gate in a three-dimensional perspective. The coordinates ${\;p}_{a}$ and ${\;p}_{b}$ represent the probabilities of logic 0 at the input A and B of XOR gate, respectively. The four blue corners below Fig. 3b indicate that two inputs of XOR gate remain unchanged when ${\;p}_{a}$ and ${\;p}_{b}$ approach either 0 or 1. These results imply that the mutual information of XOR gate approaches 0. The mutual information of XOR gate increases as ${\;p}_{a}$ and ${\;p}_{b}$ approach 0.5. Fig. 3c shows the heatmap of the mutual information of XOR gate projected onto the ${\;p}_{a}$–${\;p}_{b}$ plane. The red of thermodynamic grid is increased as ${\;p}_{a}$ and ${\;p}_{b}$ approach the central point, i.e. 0.5. The mutual information of XOR gate increases with the increase of red of thermodynamic grid.

By iterating through all possible input and output of XOR gate, the information capacity of XOR gate is given by


(7) $$C_{\mathrm{XOR}}=\max I\left(AB;Y_{\mathrm{XOR}}\right),$$

where $I(AB;Y_{\mathrm{XOR}})$ is the mutual information between the input and output of XOR gate for one operation. Supplementary Material, Appendix S3 includes the derivation of the information capacity of XOR gate. Equation 7 indicates that the maximum information rate can be achieved by transmitting information with an arbitrarily small error rate.

## Information energy ratio of XOR gates

To reveal the relationship between the information capacity and energy consumption of XOR gates at the mesoscopic scale, a new analysis method, i.e. an information energy ratio of XOR gates, is established by


(8) $$\eta_{\mathrm{XOR}}=I\left(AB;Y_{\mathrm{XOR}}\right)/{\bar{E}}_{\mathrm{XOR}}^{\mathrm{diss}}\;(bits/kT).$$

$\eta_{\mathrm{XOR}}$ refers to the number of bits processed per unit of *kT* energy at an XOR gate. A larger information energy ratio indicates that more information can be processed per *kT* energy. See Supplementary Material, Appendix S4 for details of the information energy ratio of XOR gates.

The transformation ratio between the mutual information and energy consumption of XOR gates is first quantified by 8, which can be used to analyze the impact of system parameters, e.g. the supply voltage on the information energy ratio of XOR gates.

**Fig. 4.**
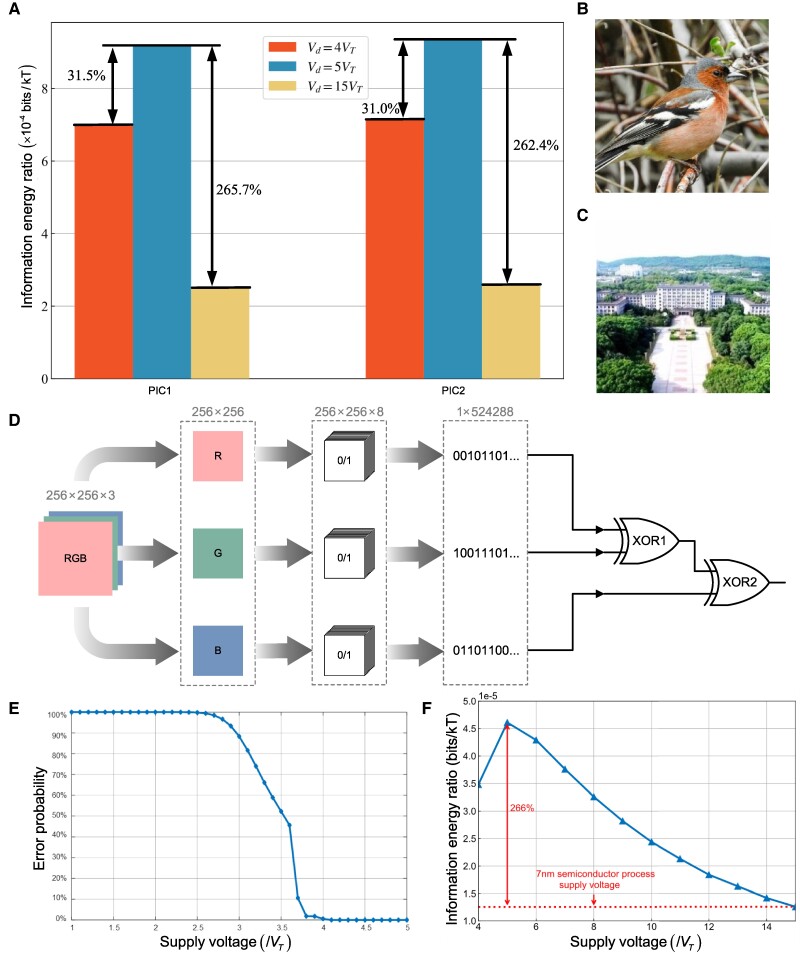
Information energy ratio and error probability of XOR gates with respect to the supply voltage. a) The information energy ratio of XOR gates operating with supply voltages $4V_{T}$, $5V_{T}$, and $15V_{T}$ when operating picture 1 and picture 2. b) Picture 1. c) Picture 2. d) The preprocessing of pictures. e) The error probability of XOR gates with respect to the supply voltage when the previous input is $a_{n-1}b_{n-1}=00$. f) The information energy ratio of the parity check circuit with respect to the supply voltage.

Without loss of generality, two pictures are selected to analyze the information energy ratio of XOR gates. Fig. 4 illustrates the information energy ratio with respect to the supply voltage for an XOR gate. Fig. 4a compares the information energy ratio of XOR gates operated with different supply voltages, i.e. 4$V_{T}$, 5$V_{T}$, and 15$V_{T}$. Fig. 4b and c is pictures for comparison. Three-dimensional, Red Green Blue (RGB) pixel points of the picture are converted into three binary bit streams, i.e. RGB binary bit streams, which are sequentially input into the parity check circuit, as shown in Fig. 4d. Compared with the information energy ratio with the supply voltage $4V_{T}$, the information energy ratio with the supply voltage $5V_{T}$ is improved by 31.5% and 31% for the pictures of Fig. 4b and 4c, respectively. Compared with the information ratio with the supply voltage $15V_{T}$, the information energy ratio with the supply voltage $5V_{T}$ is improved by 265.7% and 262.4% for the pictures of Fig. 4b and Fig. 4c, respectively.

The optimization problem to maximize the information energy ratio of XOR gates is expressed as


(9) $$\begin{array}{rl} & \;\max\eta_{\mathrm{XOR}}=f\left(V_{d},\;{\;p}_{a},\;{\;p}_{b}\right)\\ & s.t.\text{ 4}V_{T}\leq V_{d}\leq 6V_{T},\;0\leq {\;p}_{a}\leq 1,\;0\leq {\;p}_{b}\leq 1 \end{array},$$

where f(⋅) is the function based on 8. Based on the results of Fig. 4d, the range of supply voltage $V_{d}$ is configured as $\text{4}V_{T}\leq V_{d}\leq 6V_{T}$ for maximizing the information energy ratio of XOR gates. Since 8 is a discontinuous function, the Genetic Algorithm (20) (GA) is adopted to obtain the upper bound of the information energy ratio of XOR gates. In GA, the binary coding is used to encode individuals and then each individual can be regarded as a solution to the above problem (21). In this case, each individual owns a set of chromosomes represented by three variables $V_{d}$, ${\;p}_{a}$, and ${\;p}_{b}$.

The simulation parameters are configured as follows: the length of gene code is 10, the size of population is 80, the evolution times are 100, and the mutation probability is 0.001. The maximum information energy ratio is configured as the fitness function of GA. The upper bound of the information energy ratio can be achieved for XOR gates during continuous iterations. Based on simulation results, we can observe that the upper bound of information energy ratio of XOR gates converges to 0.00026 bits/kT when $V_{d}\approx 5.16V_{T}$, ${\;p}_{a}\approx 0.39$ and ${\;p}_{b}\approx 1$, i.e. the *kT* energy can maximally process 0.00026 bits of information for an XOR gate.

## Information energy ratio of parity check circuit

The information energy ratio of parity check circuit is proposed as


(10) $$\eta_{\mathrm{parity}}=I\left(ABC;Y_{\mathrm{parity}}\right)/{\bar{E}}_{\mathrm{parity}}^{\mathrm{diss}},$$

where $I(ABC;Y_{\mathrm{parity}})$ is the mutual information between the input and output of the parity check circuit, ${\bar{E}}_{\mathrm{parity}}^{\mathrm{diss}}$ is the average energy consumption for one operation of the parity check circuit, which is composed of the energy consumption of two XOR gates. See Supplementary Material, Appendix S5 for details of the information energy ratio of the parity check circuit.

The supply voltage of XOR gate is the key factor to determine the error probability of XOR gate. Moreover, the information energy ratio of the parity check circuit depends on the optimization of the supply voltage. Without loss of generality, when the previous input is $a_{n-1}b_{n-1}=00$, the error probability of XOR gates with respect to the supply voltage is simulated in Fig. 4e. Simulation results of Fig. 4e show that the error probability of XOR gates is larger than 1% when the supply voltage is less than $4V_{T}$. When the supply voltage is larger than or equal to $4V_{T}$, the error probability of XOR gates is less than or equal to 1%. Therefore, the XOR gate can function well only if the supply voltage is larger than or equal to 4$V_{T}$ considering that the error probability is required to be less than 1% in many logic operation circuit applications (22).

Based on the results of Fig. 4e, the supply voltage of XOR gates should be larger than or equal to $4V_{T}$. When the technology of transistors is 7 nm, the supply voltage of XOR gates is 0.39 Volt (23), i.e. $15V_{T}$. Without loss of generality, the range of supply voltage is configured from $4V_{T}$ to $15V_{T}$ for the parity check circuit in this article. Compared with the information energy ratio of the parity check circuit with 7 nm semiconductor process supply voltage, i.e. $15V_{T}$, results of Fig. 4f show that the information energy ratio of the parity check circuit with the supply voltage $5V_{T}$ is improved by 266%.

## Conclusions

An information and energy analysis method based on the stochastic thermodynamic is first proposed for revealing the transformation ratio between the information capacity and energy consumption of XOR gates at the mesoscopic scale. Based on the information theory and stochastic thermodynamic, the new analysis method, i.e. the information energy ratio of XOR gates, is established to quantify the transformation ratio between the information capacity and energy consumption at the mesoscopic scale. Furthermore, the information energy ratio of typical digital signal processing circuits, i.e. the parity check circuit, is proposed. Compared with the information energy ratio of the parity check circuit adopting the supply voltage at 7 nm semiconductor process, i.e. $15V_{T}$, simulation results show that the information energy ratio of the parity check circuit with the supply voltage $5V_{T}$ can be improved by 266%. Utilizing the proposed analysis method, the energy consumption of digital signal process circuits can be optimized by numerical simulations without expensive and complex practical measurements. Based on the needs of practical application, engineers can easy adjust the supply voltage to analyse the information energy ratio of digital circuits. In future work, we plan to explore new mechanisms to improve the information energy ratio of complex digital signal processing circuits.

## Materials and methods

All of our results were achieved through numerical simulations. The properties of the distribution of the output voltage are observed by both an iterative and numerically diagonalization of the master equation. The Gillespie algorithm is realized by code. Based on simulation results, the voltage of output is governed by a Gaussian distribution. The mutual information of XOR gates is given based on the information theory. Genetic Algorithm is utilized to obtain the upper bound of the information energy ratio of XOR gates. All codes are performed on PyCharm and MATLAB.

## Supplementary Material

pgae365_Supplementary_Data

## Data Availability

All data are included in the manuscript and/or supporting information. Previously published data were used for this work. The data citation is github.com/Parallel1227/An-Energy-and-Information-Analysis-Method-of-Logic-Gates-Based-on-Stochastic-Thermodynamics.git (2023).
