# Supplementary material for: An energy and information analysis method of logic gates based on stochastic thermodynamics
Source: PNAS Nexus. 2024 Aug 26;3(9):pgae365. doi: 10.1093/pnasnexus/pgae365 (PMC11410042; doi:10.1093/pnasnexus/pgae365)
Supplement: pgae365_Supplementary_Data [file pgae365_supplementary_data.zip › PNASNEXUS-PNASNEXUS-2024-00048-TRR-s01.pdf]

Supporting Information for

An Energy and Information Analysis Method of Logic Gates Based on Stochastic Thermodynamics

Xiaohu Ge, Muyao Ruan, Xiaoxuan Peng, Yong Xiao and Yang Yang

Yong Xiao and Yang Yang.
E-mail: yongxiao@hust.edu.cn, yyiot@hkust-gz.edu.cn

This PDF file includes:

Supporting text
SI References

Supporting Information Text

Appendix S1: Detailed energy consumption derivation of NAND gate

In this appendix, the details about the energy consumption derivation of NAND gate are given. At the moment t, the state distribution of NAND gate is represented by the electron number distribution of transistors, i.e., $\mathsf{s}^t = [n_{\mathrm{N}_1}, n_{\mathrm{N}_2}, n_{\mathrm{P}_1}, n_{\mathrm{P}_2}]$, where n_{N_1} and n_{N_2} represent the numbers of electrons in the N-type transistors N_1 and N_2, n_{P_1} and n_{P_2} represent the number of electrons in the P-type transistors P_1 and P_2, respectively. Considering the single-electron transistor adopted in this article, the range of electron number is configured as $n_{\mathrm{N}_1}, n_{\mathrm{N}_2}, n_{\mathrm{P}_1}, n_{\mathrm{P}_2} \in \{0, 1\}$ for a transistor.

When the local detailed balance condition can be satisfied (1), the transfer process of electrons within the logic gate can be approximated as a Markovian process. Let $\mathsf{s}^{t+\Delta t}$ be the state distribution of NAND gates at the moment $t + \Delta t$. The state transition of NAND gate is given by

$$\mathsf{s}^{t+\Delta t} = \mathbf{D}_{\mathrm{NAND}} \cdot \mathsf{s}^t, \tag{1}$$

where $\mathbf{D}_{\mathrm{NAND}}$ is the transition rate matrix of NAND gate, which is described as (2)

$$\mathbf{D}_{\mathrm{NAND}} = \begin{bmatrix}
-F_1 & \delta_{\mathrm{dP}_1} + \delta_{\mathrm{gP}_1} & \cdots & 0 & 0 \\
\delta_{\mathrm{P}_1\mathrm{d}} + \delta_{\mathrm{P}_1\mathrm{g}} & -F_2 & \cdots & 0 & 0 \\
\delta_{\mathrm{P}_2\mathrm{d}} + \delta_{\mathrm{P}_2\mathrm{g}} & \delta_{\mathrm{P}_2\mathrm{P}_1} & \cdots & 0 & 0 \\
\delta_{\mathrm{N}_1\mathrm{g}} & \delta_{\mathrm{N}_1\mathrm{P}_1} & \cdots & 0 & 0 \\
\delta_{\mathrm{N}_2\mathrm{s}} & 0 & \cdots & 0 & 0 \\
0 & \delta_{\mathrm{P}_2\mathrm{d}} + \delta_{\mathrm{P}_2\mathrm{g}} & \cdots & \delta_{\mathrm{gN}_1} & 0 \\
0 & \delta_{\mathrm{N}_1\mathrm{g}} & \cdots & \delta_{\mathrm{dP}_2} + \delta_{\mathrm{gP}_2} & 0 \\
0 & \delta_{\mathrm{N}_2\mathrm{s}} & \cdots & 0 & 0 \\
0 & 0 & \cdots & \delta_{\mathrm{dP}_1} + \delta_{\mathrm{gP}_1} & 0 \\
0 & 0 & \cdots & 0 & 0 \\
0 & 0 & \cdots & 0 & 0 \\
0 & 0 & \cdots & 0 & \delta_{\mathrm{dP}_1} + \delta_{\mathrm{gP}_1} \\
0 & 0 & \cdots & 0 & \delta_{\mathrm{dP}_2} + \delta_{\mathrm{gP}_2} \\
0 & 0 & \cdots & \delta_{\mathrm{N}_2\mathrm{N}_1} & \delta_{\mathrm{gN}_1} \\
0 & 0 & \cdots & -F_{14} & \delta_{\mathrm{sN}_2} \\
0 & 0 & \cdots & \delta_{\mathrm{N}_2\mathrm{s}} & -F_{15}
\end{bmatrix}, \tag{2}$$

where $F_n = \sum_{m \neq n} (\mathbf{D}_{\mathrm{NAND}})_{m,n}$. Transfer rates between electrode i and transistor j are denoted as $\delta_{ji} = \Gamma \cdot f_i(\varepsilon_j)$ and $\delta_{ij} = \Gamma \cdot [1 - f_i(\varepsilon_j)]$, respectively. The function $f_i(\varepsilon_j)$ is a Fermi distribution and denoted by $f_i(\varepsilon_j) = \left[e^{\beta(\varepsilon_j - \mu_i)} + 1\right]^{-1}$. μ_i and ε_j are the chemical potential of the electrode i and the energy level of the transistor j, respectively. μ_i and ε_j have a linear relationship with the voltage (3). By adjusting the linear relationship between the energy level and voltage, characteristics of different types of transistors can be mimicked. Γ is a rate constant. $\beta = 1/kT$, where k is the Boltzmann coefficient, T is the temperature. The transfer rate between the transistors j_1 and j_2 follows the Bose-Einstein distribution, $j_1, j_2 \in \{\mathrm{P}_1, \mathrm{P}_2, \mathrm{N}_1, \mathrm{N}_2\}$.

Based on the transition rate matrix $\mathbf{D}_{\mathrm{NAND}}$, the average electron amount $\langle n_j \rangle$ of the transistor j can be obtained at the present observation time interval. Furthermore, the current flowing from the electrode i to the transistor j is denoted as $J_{i \to j}$, which is calculated by

$$J_{i \to j} = q\left[\delta_{ji}\left(1 - \langle n_j \rangle\right) - \delta_{ij}\langle n_j \rangle\right], \tag{3}$$

where q is the electric charge unit, $\langle n_j \rangle$ is the average number of electrons of the transistor j at the present observation time. Furthermore, the energy consumption of gate NAND_r is given by

$$W_{\mathrm{NAND}}^r(\tau) = \int_0^\tau \left[J_{\mathrm{s} \to \mathrm{N}_2}\left(\mu_{\mathrm{s}} - \mu_{\mathrm{g}}\right) + J_{\mathrm{d} \to \mathrm{P}_1}\left(\mu_{\mathrm{d}} - \mu_{\mathrm{g}}\right) + J_{\mathrm{d} \to \mathrm{P}_2}\left(\mu_{\mathrm{d}} - \mu_{\mathrm{g}}\right)\right] dt, \tag{4}$$

where the upper bound of integration τ is the propagation delay, which is taken as the moment when the difference between the output voltage and the expected voltage exceeds a certain threshold. Eq. (4) shows that the energy consumption of gate NAND_r results from the continuous migration of electrons between transistors and electrodes.

Appendix S2: Detailed mutual information derivation of XOR gate

In this appendix, the details about the mutual information between the input and output of XOR gate are given. Based on the stochastic thermodynamic model of XOR gate, the output voltage distribution of XOR gate is governed by Gaussian

distributions based on Gillespie's algorithm (3). Assume that the output symbol of XOR gate is y_n^{XOR} at the time step n. The probability density function of output voltage of XOR gate $V_{\text{out}}^{\text{XOR}}$ is expressed as

$$f_{\text{p}}\left(V_{\text{out}}^{\text{XOR}}\right)=\begin{cases}\sqrt{\frac{\beta C_{\text{g}}}{2\pi}}\exp\left[-\frac{1}{2}\beta C_{\text{g}}\left(V_{\text{out}}^{\text{XOR}}\right)^2\right]\times\mathbb{I}_{+}\text{ , if } a_n\oplus b_n=0\\ \sqrt{\frac{\beta C_{\text{g}}}{2\pi}}\exp\left[-\frac{1}{2}\beta C_{\text{g}}\left(V_{\text{out}}^{\text{XOR}}-V_{\text{d}}\right)^2\right]\times\mathbb{I}_{-}\text{, else}\end{cases},\quad[5]$$

where $\mathbb{I}_{+}=1$ when $V_{\text{out}}^{\text{XOR}}\geq 0$, otherwise $\mathbb{I}_{+}=0$. $\mathbb{I}_{-}=1$ when $V_{\text{out}}^{\text{XOR}}\leq V_{\text{d}}$, otherwise $\mathbb{I}_{-}=0$. The symbol $\oplus$ denotes the operation of XOR. Therefore, the mutual information between the input and output of XOR gate is derived as

$$\begin{aligned}I\left(AB;Y_{\text{XOR}}\right)&=\sum_{a_n\in A}\sum_{b_n\in B}\sum_{y_n^{\text{XOR}}\in Y_{\text{XOR}}}p\left(a_n,b_n,y_n^{\text{XOR}}\right)\log_2\left(\frac{p\left(a_n,b_n,y_n^{\text{XOR}}\right)}{p\left(a_nb_n\right)p\left(y_n^{\text{XOR}}\right)}\right)\\&=\sum_{a_n\in A}\sum_{b_n\in B}\sum_{y_n^{\text{XOR}}\in Y_{\text{XOR}}}p\left(a_n,b_n,y_n^{\text{XOR}}\right)\log_2\left(\frac{p\left(y_n^{\text{XOR}}|a_nb_n\right)}{p\left(y_n^{\text{XOR}}\right)}\right)\end{aligned},\quad[6]$$

where $p\left(a_n,b_n,y_n^{\text{XOR}}\right)$ is the joint probability when the input of XOR gate is a_nb_n and the output symbol is y_n^{XOR}, $p\left(a_nb_n\right)$ is the probability when the input is a_nb_n, $p\left(y_n^{\text{XOR}}|a_nb_n\right)$ is the conditional probability when the output symbol is y_n^{XOR} and the input symbol is a_nb_n, and $p\left(y_n^{\text{XOR}}\right)$ is the probability when the output symbol is y_n^{XOR}.

Appendix S3: Detailed information capacity of XOR gate

In this appendix, the details about the information capacity of XOR gate are given. Based on the schematic diagram of information processing process, the amount of information at the input and output are expressed as follows

$$I_{\text{in}}=\sum_{x_{\text{in}}\in\mathcal{X}}p\left(x_{\text{in}}\right)\log_2\left(\frac{1}{p\left(x_{\text{in}}\right)}\right),\quad[7]$$

$$I_{\text{out}}=\sum_{y_{\text{out}}\in\mathcal{Y}}p\left(y_{\text{out}}\right)\log_2\left(\frac{1}{p\left(y_{\text{out}}\right)}\right),\quad[8]$$

where $p\left(x_{\text{in}}\right)$ is the probability when the input symbol is x_{in}, $p\left(y_{\text{out}}\right)$ is the probability when the output symbol is y_{out}.

Considering all possible input states, we denote the noise N of a process as the uncertainty in the output given the input state

$$N=\sum_{x_{\text{in}}\in\mathcal{X}}p\left(x_{\text{in}}\right)\sum_{y_{\text{out}}\in\mathcal{Y}}p\left(y_{\text{out}}|x_{\text{in}}\right)\log_2\left(\frac{1}{p\left(y_{\text{out}}|x_{\text{in}}\right)}\right),\quad[9]$$

where $p\left(y_{\text{out}}|x_{\text{in}}\right)$ is the conditional probability of output y_{out} given the input state x_{in}. The mutual information between the input and output is expressed as

$$\Omega=I_{\text{out}}-N.\quad[10]$$

Based on Eq. (10), the mutual information between the input and output of XOR gate is obtained. The maximum of mutual information is the information capacity of XOR gate for a single operation.

The input states of XOR gate are classified into 4 cases: $p\left(y_n^{\text{XOR}}=0|a_nb_n\in\{00,11\}\right)$, $p\left(y_n^{\text{XOR}}=1|a_nb_n\in\{01,10\}\right)$, $p\left(y_n^{\text{XOR}}=0|a_nb_n\in\{01,10\}\right)$, $p\left(y_n^{\text{XOR}}=1|a_nb_n\in\{00,11\}\right)$. When $a_nb_n\in\{00,11\}$, the error probability is expressed as

$$\xi_{a_nb_n\in\{00,11\}}=p\left(y_n^{\text{XOR}}\neq 0|a_nb_n\in\{00,11\}\right)=\Phi\left(\frac{a_nb_n}{\sigma}\right)\approx 1-\frac{1}{2\sqrt{\pi\beta C_{\text{g}}}}\frac{e^{-\beta C_{\text{g}}(1-\alpha)^2{V_{\text{d}}}^2}}{(1-\alpha)V_{\text{d}}},\quad[11]$$

where the function Φ is the cumulative normal distribution function of standard Gaussian distribution, which is denoted as $\Phi\left(\omega\right)=\frac{1}{\sqrt{2\pi}}\int_{-\infty}^{\omega}e^{-\theta^2/2}d\theta$, where $\omega=a_nb_n/\sigma$, $\sigma=\sqrt{1/\beta C_{\text{g}}}$. When $a_nb_n\in\{01,10\}$, the error probability is expressed as

$$\xi_{a_nb_n\in\{01,10\}}=p\left(y_n^{XOR}\neq 1|a_nb_n\in\{01,10\}\right)=\Phi\left(\frac{a_nb_n-V_{\text{d}}}{\sigma}\right)=\frac{1}{2\sqrt{\pi\beta C_{\text{g}}}}\frac{e^{-\beta C_{\text{g}}\alpha^2{V_{\text{d}}}^2}}{\alpha V_{\text{d}}}.\quad[12]$$

Based on Eq. (11) and Eq. (12), the transition probability of XOR gate is derived as

$$\begin{cases} p\left(y_n^{\mathrm{XOR}}=0|a_nb_n\in\{01,10\}\right)=\xi_{a_nb_n\in\{00,11\}} \\ p\left(y_n^{\mathrm{XOR}}=1|a_nb_n\in\{01,10\}\right)=1-\xi_{a_nb_n\in\{00,11\}} \\ p\left(y_n^{\mathrm{XOR}}=0|a_nb_n\in\{00,11\}\right)=1-\xi_{a_nb_n\in\{01,10\}} \\ p\left(y_n^{\mathrm{XOR}}=1|a_nb_n\in\{00,11\}\right)=\xi_{a_nb_n\in\{01,10\}} \end{cases}. \quad [13]$$

The information capacity of XOR gate for a single operation is given by

$$\begin{aligned} C_{\mathrm{XOR}} &= \max I\left(AB;Y_{\mathrm{XOR}}\right) \\ &= \max \sum_{a_n\in A}\sum_{b_n\in B}\sum_{y_n^{\mathrm{XOR}}\in Y_{\mathrm{XOR}}} p\left(a_n,b_n,y_n^{\mathrm{XOR}}\right)\log_2\left(\frac{p\left(y_n^{\mathrm{XOR}}|a_nb_n\right)}{p\left(y_n^{\mathrm{XOR}}\right)}\right). \end{aligned} \quad [14]$$

Appendix S4: Detailed information energy ratio of XOR gate

In this appendix, the details about the information energy radio of XOR gate are given. The mutual information of XOR gate is derived as

$$\begin{aligned} &I\left(AB;Y_{\mathrm{XOR}}\right) \\ &= \sum_{a_n\in A}\sum_{b_n\in B}\sum_{y_n^{\mathrm{XOR}}\in Y_{\mathrm{XOR}}} p\left(a_n,b_n,y_n^{\mathrm{XOR}}\right)\log_2\frac{p\left(y_n^{\mathrm{XOR}}|a_nb_n\right)}{p\left(y_n^{\mathrm{XOR}}\right)} \\ &= \left(p_{00}+p_{11}\right)\sum_{y_n^{\mathrm{XOR}}\in Y_{\mathrm{XOR}}} p\left(y_n^{\mathrm{XOR}}|a_nb_n=00\right)\log_2\frac{p\left(y_n^{\mathrm{XOR}}|a_nb_n=00\right)}{p\left(y_n^{\mathrm{XOR}}\right)} \\ &\quad + \left(p_{01}+p_{10}\right)\sum_{y_n^{\mathrm{XOR}}\in Y_{\mathrm{XOR}}} p\left(y_n^{\mathrm{XOR}}|a_nb_n=01\right)\log_2\frac{p\left(y_n^{\mathrm{XOR}}|a_nb_n=01\right)}{p\left(y_n^{\mathrm{XOR}}\right)} \\ &= \left(\left\|P_{\mathrm{XOR}}^{\mathrm{trans}}\cdot\begin{bmatrix}1&0&0&0\end{bmatrix}^{\mathrm{T}}\right\|_1+\left\|P_{\mathrm{XOR}}^{\mathrm{trans}}\cdot\begin{bmatrix}0&0&0&1\end{bmatrix}^{\mathrm{T}}\right\|_1\right)\sum_{y_n^{\mathrm{XOR}}\in Y_{\mathrm{XOR}}} p\left(y_n^{\mathrm{XOR}}|a_nb_n=00\right)\log_2\frac{p\left(y_n^{\mathrm{XOR}}|a_nb_n=00\right)}{p\left(y_n^{\mathrm{XOR}}\right)} \\ &\quad + \left(\left\|P_{\mathrm{XOR}}^{\mathrm{trans}}\cdot\begin{bmatrix}0&1&0&0\end{bmatrix}^{\mathrm{T}}\right\|_1+\left\|P_{\mathrm{XOR}}^{\mathrm{trans}}\cdot\begin{bmatrix}0&0&1&0\end{bmatrix}^{\mathrm{T}}\right\|_1\right)\sum_{y_n^{\mathrm{XOR}}\in Y_{\mathrm{XOR}}} p\left(y_n^{\mathrm{XOR}}|a_nb_n=01\right)\log_2\frac{p\left(y_n^{\mathrm{XOR}}|a_nb_n=01\right)}{p\left(y_n^{\mathrm{XOR}}\right)} \end{aligned}, \quad [15]$$

where p_{ab} is the probability when the input symbols of XOR gate are a_n and b_n at the time step n. The norm of vector l is $\|l\|_1=\sum_{i=1}^{n}|l_i|$. Superscript T is a transposing operation for a matrix. Based on Eq. (15), the information energy radio of XOR gate can be rewritten in detail as

$$\eta_{\mathrm{XOR}} = \frac{\left[\begin{aligned} &\left(\left\|\mathbf{P}_{\mathrm{XOR}}^{\mathrm{trans}}\cdot\begin{bmatrix}1&0&0&0\end{bmatrix}^{\mathrm{T}}\right\|_1+\left\|\mathbf{P}_{\mathrm{XOR}}^{\mathrm{trans}}\cdot\begin{bmatrix}0&0&0&1\end{bmatrix}^{\mathrm{T}}\right\|_1\right)\sum_{y_n^{\mathrm{XOR}}\in Y_{\mathrm{XOR}}}\left(p\left(y_n^{\mathrm{XOR}}|a_nb_n=00\right)\log_2\left(\frac{p\left(y_n^{\mathrm{XOR}}|a_nb_n=00\right)}{p\left(y_n^{\mathrm{XOR}}\right)}\right)\right)+ \\ &\left(\left\|\mathbf{P}_{\mathrm{XOR}}^{\mathrm{trans}}\cdot\begin{bmatrix}0&1&0&0\end{bmatrix}^{\mathrm{T}}\right\|_1+\left\|\mathbf{P}_{\mathrm{XOR}}^{\mathrm{trans}}\cdot\begin{bmatrix}0&0&1&0\end{bmatrix}^{\mathrm{T}}\right\|_1\right)\sum_{y_n^{\mathrm{XOR}}\in Y_{\mathrm{XOR}}}\left(p\left(y_n^{\mathrm{XOR}}|a_nb_n=01\right)\log_2\left(\frac{p\left(y_n^{\mathrm{XOR}}|a_nb_n=01\right)}{p\left(y_n^{\mathrm{XOR}}\right)}\right)\right) \end{aligned}\right]}{\sum_{a_{n-1}b_{n-1}}\sum_{a_nb_n}\begin{pmatrix} E_{00\to00}p_{00\to00} & E_{00\to01}p_{00\to01} & E_{00\to10}p_{00\to10} & E_{00\to11}p_{00\to11} \\ E_{01\to00}p_{01\to00} & E_{01\to01}p_{01\to01} & E_{01\to10}p_{01\to10} & E_{01\to11}p_{01\to11} \\ E_{10\to00}p_{10\to00} & E_{10\to01}p_{10\to01} & E_{10\to10}p_{10\to10} & E_{10\to11}p_{10\to11} \\ E_{11\to00}p_{11\to00} & E_{11\to01}p_{11\to01} & E_{11\to10}p_{11\to10} & E_{11\to11}p_{11\to11} \end{pmatrix}_{a_{n-1}b_{n-1},a_nb_n}}. \quad [16]$$

Appendix S5: Detailed information energy radio of parity check circuit

In this appendix, the details about the information energy radio of parity check circuit are given. Based on the information energy ratio of XOR gate, the information energy ratio of parity check circuit is investigated. Parity check circuits perform the function of detecting the number of logic 1 among three input symbols of the circuit.

The sequences of three input symbols with length M are represented as $S_A = \{a_1, ...a_{n-1}, a_n, ..., a_M\}$, $S_B = \{b_1, ...b_{n-1}, b_n, ..., b_M\}$ and $S_C = \{c_1, ...c_{n-1}, c_n, ..., c_M\}$, through the input A, B and C. a_n, b_n and c_n represent the three input symbols at the time step n with $a_n b_n c_n \in \{000, 001, 010, 011, 100, 101, 110, 111\}$. Suppose that three inputs of the parity check circuit are independent identity distributions (i.i.d.). Based on the mutual information of XOR gate, the mutual information between the input and output of parity check circuit at the time step n is

$$\begin{aligned} I\left(ABC; Y_{\text{parity}}\right) &= \sum_{a_n\in A}\sum_{b_n\in B}\sum_{c_n\in C}\sum_{y_n^{\text{parity}}\in Y_{\text{XOR}}} p\left(a_n, b_n, c_n, y_n^{\text{parity}}\right)\log_2\left(\frac{p\left(a_n, b_n, c_n, y_n^{\text{parity}}\right)}{p\left(a_n b_n c_n\right)p\left(y_n^{\text{parity}}\right)}\right) \\ &= \sum_{a_n\in A}\sum_{b_n\in B}\sum_{c_n\in C}\sum_{y_n^{\text{parity}}\in Y_{\text{XOR}}} p\left(a_n, b_n, c_n, y_n^{\text{parity}}\right)\log_2\left(\frac{p\left(y_n^{\text{parity}}|a_n b_n c_n\right)}{p\left(y_n^{\text{parity}}\right)}\right) \end{aligned}, \qquad [17]$$

where y_n^{parity} is the output symbol of XOR2 at the time step n, $y_n^{\text{parity}} \in Y_{\text{parity}}$. $p\left(a_n, b_n, c_n, y_n^{\text{parity}}\right)$ is the joint probability when three input symbols of parity check circuit are $a_n b_n c_n$ and the output symbol is y_n^{parity}, $p\left(a_n b_n c_n\right)$ is the probability when three input symbols are $a_n b_n c_n$, $p\left(y_n^{\text{parity}}|a_n b_n c_n\right)$ is the conditional probability when the output symbol is y_n^{parity} and three input symbols are $a_n b_n c_n$, $p\left(y_n^{\text{parity}}\right)$ is the probability when the output symbol is y_n^{parity}. For all eight cases of the inputs and three cases of the output, the mutual information between the input and output of parity check circuits can be obtained by traversing all 24 cases.

The average energy consumption of a single computing of parity check circuit is expressed as

$$\overline{E}_{\text{parity}}^{\text{diss}} = \sum_{a_{n-1}b_{n-1}c_{n-1}}\sum_{a_n b_n c_n}\left(\mathsf{E}_{\text{XOR}}^{\text{diss}}\cdot\mathsf{P}_{\text{XOR1}}^{\text{trans}} + \mathsf{E}_{\text{XOR}}^{\text{diss}}\cdot\mathsf{P}_{\text{XOR2}}^{\text{trans}}\right)_{a_{n-1}b_{n-1}c_{n-1}, a_n b_n c_n}, \qquad [18]$$

where $\mathbf{P}_{\text{XOR1}}^{\text{trans}}$ and $\mathbf{P}_{\text{XOR2}}^{\text{trans}}$ are the input state transition matrixes of XOR1 and XOR2 for a single computing of parity check circuit, which are denoted as

$$\mathbf{P}_{\text{XOR1}}^{\text{trans}} = \begin{pmatrix} {p_{a_n}}^2{p_{b_n}}^2 & {p_{a_n}}^2 p_{b_n}\overline{p_{b_n}} & p_{a_n}\overline{p_a}{p_{b_n}}^2 & p_{a_n}\overline{p_{a_n}}p_{b_n}\overline{p_{b_n}} \\ {p_{a_n}}^2 p_{b_n}\overline{p_{b_n}} & {p_{a_n}}^2\overline{p_{b_n}}^2 & p_{a_n}\overline{p_{a_n}}p_{b_n}\overline{p_{b_n}} & p_{a_n}\overline{p_{a_n}}\overline{p_{b_n}}^2 \\ p_{a_n}\overline{p_{a_n}}{p_{b_n}}^2 & p_{a_n}\overline{p_{a_n}}p_{b_n}\overline{p_{b_n}} & \overline{p_{a_n}}^2{p_{b_n}}^2 & \overline{p_{a_n}}^2 p_{b_n}\overline{p_{b_n}} \\ p_{a_n}\overline{p_{a_n}}p_{b_n}\overline{p_{b_n}} & p_{a_n}\overline{p_{a_n}}\overline{p_{b_n}}^2 & \overline{p_{a_n}}^2 p_{b_n}\overline{p_{b_n}} & \overline{p_{a_n}}^2\overline{p_{b_n}}^2 \end{pmatrix}, \qquad [19]$$

$$\mathbf{P}_{\text{XOR2}}^{\text{trans}} = \begin{pmatrix} \lambda_1{p_{c_n}}^2 & \lambda_1 p_{c_n}\overline{p_{c_n}} & \lambda_2{p_{c_n}}^2 & \lambda_2 p_{c_n}\overline{p_{c_n}} \\ \lambda_1 p_{c_n}\overline{p_{c_n}} & \lambda_1\overline{p_{c_n}}^2 & \lambda_2 p_{c_n}\overline{p_{c_n}} & \lambda_2\overline{p_{c_n}}^2 \\ \lambda_3{p_{c_n}}^2 & \lambda_3 p_{c_n}\overline{p_{c_n}} & \lambda_4{p_{c_n}}^2 & \lambda_4 p_{c_n}\overline{p_{c_n}} \\ \lambda_3 p_{c_n}\overline{p_{c_n}} & \lambda_3\overline{p_{c_n}}^2 & \lambda_4 p_{c_n}\overline{p_{c_n}} & \lambda_4\overline{p_{c_n}}^2 \end{pmatrix}, \qquad [20]$$

where each matrix element of $\mathbf{P}_{\text{XOR1}}^{\text{trans}}$ and $\mathbf{P}_{\text{XOR2}}^{\text{trans}}$ is the transition probability of the two input symbols of XOR1 and XOR2 between the time step $n-1$ and n, respectively. For simplicity, λ_1, λ_2 and λ_3 are introduced and are expressed as follows

$$\begin{cases} \lambda_1 = {p_{a_n}}^2{p_{b_n}}^2 + 2p_{a_n}\overline{p_{a_n}}p_{b_n}\overline{p_{b_n}} + \overline{p_{a_n}}^2\overline{p_{b_n}}^2 \\ \lambda_2 = {p_{a_n}}^2 p_{b_n}\overline{p_{b_n}} + p_{a_n}\overline{p_{a_n}}{p_{b_n}}^2 + p_{a_n}\overline{p_{a_n}}\overline{p_{b_n}}^2 + \overline{p_{a_n}}^2 p_b\overline{p_{b_n}} \\ \lambda_3 = {p_{a_n}}^2 p_{b_n}\overline{p_{b_n}} + p_{a_n}\overline{p_{b_n}}{p_{b_n}}^2 + p_{a_n}\overline{p_{a_n}}\overline{p_{b_n}}^2 + \overline{p_{a_n}}^2 p_{b_n}\overline{p_{b_n}} \end{cases}, \qquad [21]$$

where $\overline{p_s} = 1 - p_s$, $s \in \{a_n, b_n, c_n\}$. p_{a_n} is the probability when the input symbol a_n is logic 0, p_{b_n} is the probability when the input symbol b_n is logic 0, and p_{c_n} is the probability when the input symbol c_n is logic 0.

Furthermore, combining with Eq. (17) and Eq. (18), the information energy ratio of parity check circuit is expressed as

$$\begin{aligned} \eta_{\text{parity}} &= I\left(ABC; Y_{\text{parity}}\right)/\overline{E}_{\text{parity}}^{\text{diss}} \\ &= \frac{\sum\limits_{a_n\in A}\sum\limits_{b_n\in B}\sum\limits_{c_n\in C}\sum\limits_{y_n^{\text{parity}}\in Y_{\text{XOR}}} p\left(a_n, b_n, c_n, y_n^{\text{parity}}\right)\log_2\left(\frac{p\left(y_n^{\text{parity}}|a_n b_n c_n\right)}{p\left(y_n^{\text{parity}}\right)}\right)}{\sum\limits_{a_{n-1}b_{n-1}c_{n-1}}\sum\limits_{a_n b_n c_n}\left(\mathbf{E}_{\text{XOR}}^{\text{diss}}\cdot\mathbf{P}_{\text{XOR1}}^{\text{trans}} + E_{\text{XOR}}^{\text{diss}}\cdot P_{\text{XOR2}}^{\text{trans}}\right)_{a_{n-1}b_{n-1}c_{n-1}, a_n b_n c_n}} \end{aligned}. \qquad [22]$$

References

1. FS Gnesotto, F Mura, J Gladrow, CP Broedersz, Broken detailed balance and non-equilibrium dynamics in living systems: a review. *Reports on Prog. Phys.* **81**, 066601 (2018).
2. J Kuang, X Ge, Y Yang, L Tian, Modeling and optimization of low-power and gates based on stochastic thermodynamics. *IEEE Transactions on Circuits Syst. II: Express Briefs* **69**, 3729–3733 (2022).
3. Y Gao, *Molecular Insight into Nonlinear Transport Behaviors.* (2021).
